# Tetrakis[μ_3_-2-(piperidin-1-yl)ethanolato]tetrakis[chloridocopper(II)]

**DOI:** 10.1107/S1600536814009052

**Published:** 2014-04-30

**Authors:** Mei Luo, Yong-Hua Huang, Jing-Cheng Zhang

**Affiliations:** a Hefei University of Technology, Hefei, People’s Republic of China

## Abstract

In the title tetra­nuclear compound, [Cu_4_(C_7_H_14_NO)_4_Cl_4_], each Cu^II^ cation is *N*,*O*-chelated by a piperidineethanolate anion and coordinated by a Cl^−^ anion and two O atoms from neighboring piperidine­ethano­late anions in a distorted NO_3_Cl square-pyramidal geometry. The deprotonated hydroxyl groups of the piperidineethanolate anions bridge Cu^II^ cations, forming the tetra­nuclear complex. All piperidine rings display a chair conformation. In the crystal, there are no significant inter­molecular inter­actions present. The crystal studied was an inversion twin refined with a minor component of 0.18 (5).

## Related literature   

For related metal complexes with piperidineethanolate as a chelating ligand, see: Yilmaz *et al.* (2010 ▶); Hamamci *et al.* (2008 ▶).
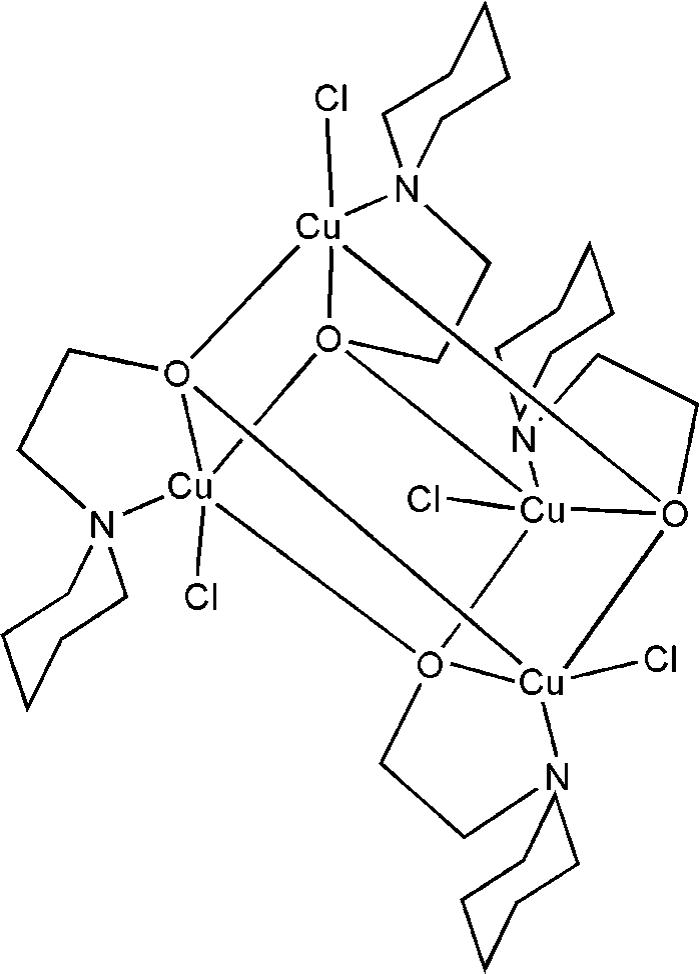


## Experimental   

### 

#### Crystal data  

[Cu_4_(C_7_H_14_NO)_4_Cl_4_]
*M* *_r_* = 908.72
Tetragonal, 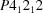

*a* = 13.9016 (2) Å
*c* = 38.8340 (9) Å
*V* = 7504.8 (3) Å^3^
*Z* = 8
Cu *K*α radiation
μ = 5.47 mm^−1^
*T* = 293 K
0.16 × 0.12 × 0.08 mm

#### Data collection  

Bruker APEXII CCD diffractometer
Absorption correction: multi-scan (*SADABS*; Bruker, 2001 ▶) *T*_min_ = 0.569, *T*_max_ = 0.753
66705 measured reflections
6976 independent reflections
6547 reflections with *I* > 2σ(*I*)
*R*_int_ = 0.066

#### Refinement  

*R*[*F* ^2^ > 2σ(*F* ^2^)] = 0.068
*wR*(*F* ^2^) = 0.182
*S* = 1.17
6976 reflections
398 parameters
H-atom parameters constrained
Δρ_max_ = 0.93 e Å^−3^
Δρ_min_ = −0.81 e Å^−3^
Absolute structure: Flack (1983 ▶), 2913 Friedel pairs
Absolute structure parameter: 0.18 (5)

### 

Data collection: *APEX2* (Bruker, 2007 ▶); cell refinement: *SAINT* (Bruker, 2007 ▶); data reduction: *SAINT*; program(s) used to solve structure: *SHELXTL* (Sheldrick, 2008 ▶); program(s) used to refine structure: *SHELXTL*; molecular graphics: *SHELXTL*; software used to prepare material for publication: *SHELXTL*.

## Supplementary Material

Crystal structure: contains datablock(s) I, global. DOI: 10.1107/S1600536814009052/xu5784sup1.cif

Structure factors: contains datablock(s) I. DOI: 10.1107/S1600536814009052/xu5784Isup2.hkl

Supporting information file. DOI: 10.1107/S1600536814009052/xu5784Isup3.cdx

CCDC reference: 998731

Additional supporting information:  crystallographic information; 3D view; checkCIF report

## Figures and Tables

**Fig. 1.**
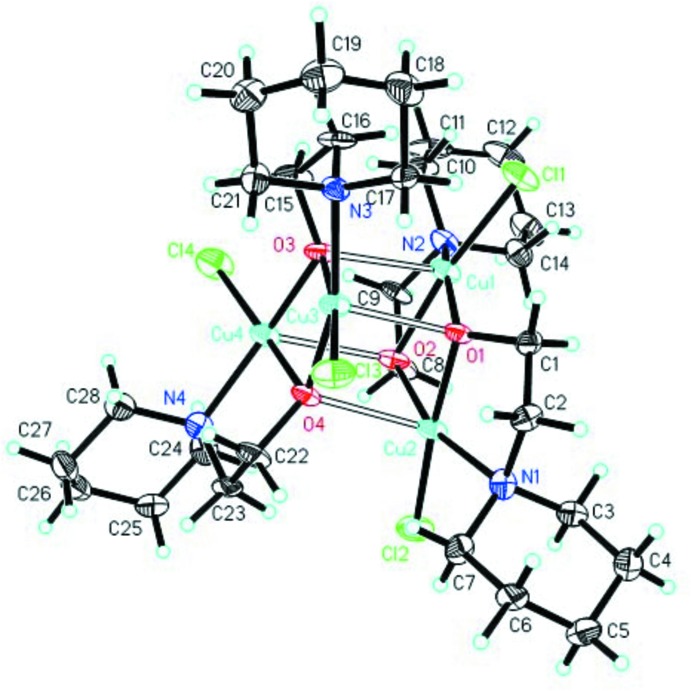
The structure of the title compound with displacement ellipsoids drawn at the 50% probability level.

## supplementary crystallographic information

### 1. Comment

The piperidineethanol chelated metal complexes have been synthesized and their crystal structures have been reported previously (Hamamci *et al.*, 2008; Yilmaz *et al.* 2010). We report here the structure of the title tetranuclear complex, a Cu^II^ complex N,O-chelated by the deprotonated piperidineethanol anion.

The molecular structure is shown in Fig. 1. Each Cu^II^ cation is N,O-chelated by a piperidineethnolate anion and coordinated by a Cl^-^ anion and two O atoms from neighboring piperidineethanolate anions in a distorted NO_3_Cl square-pyramidal geometry. The Cu—O bond distances in the apical direction are significantly longer than those in the basal planes. The deprotonated hydroxyl groups of piperidineethanolate anions bridge the Cu^II^ cations to form the tetranuclear complex. The piperidine ring displays a chair conformation.

In the crystal, there are no significant intermolecular interactions present.

### 2. Experimental

1-(2-Hydroxyethyl)piperidine (1.292 g, 10 mmol) was added to a methanol solution (50 ml) of CuCl_2_.2H_2_O (0.853 g, 5 mmol). The mixture was refluxed for 48 h. The filtrate was slowly evaporated, the blue single crystals were obtained after a day.

### 3. Refinement

H atoms were placed in calculated positions with C—H = 0.97 Å, and refined in riding mode with U_iso_(H) = 1.2U_eq_(C).

### Crystal data

**Table:**

[Cu_4_(C_7_H_14_NO)_4_Cl_4_]	*D*_x_ = 1.609 Mg m^−^^3^
*M**_r_* = 908.72	Cu *K*α radiation, λ = 1.54178 Å
Tetragonal, *P*4_1_2_1_2	Cell parameters from 9940 reflections
Hall symbol: P 4abw 2nw	θ = 3.9–68.8°
*a* = 13.9016 (2) Å	µ = 5.47 mm^−^^1^
*c* = 38.8340 (9) Å	*T* = 293 K
*V* = 7504.8 (3) Å^3^	Prism, blue
*Z* = 8	0.16 × 0.12 × 0.08 mm
*F*(000) = 3744	

### Data collection

**Table:**

Bruker APEXII CCD diffractometer	6547 reflections with *I* > 2σ(*I*)
φ and ω scans	*R*_int_ = 0.066
Absorption correction: multi-scan (*SADABS*; Bruker, 2001)	θ_max_ = 69.7°, θ_min_ = 3.4°
*T*_min_ = 0.569, *T*_max_ = 0.753	*h* = −16→16
66705 measured reflections	*k* = −16→15
6976 independent reflections	*l* = −45→46

### Refinement

**Table:**

Refinement on *F*^2^	Secondary atom site location: difference Fourier map
Least-squares matrix: full	Hydrogen site location: inferred from neighbouring sites
*R*[*F*^2^ > 2σ(*F*^2^)] = 0.068	H-atom parameters constrained
*wR*(*F*^2^) = 0.182	*w* = 1/[σ^2^(*F*_o_^2^) + (0.0407*P*)^2^ + 55.9102*P*] where *P* = (*F*_o_^2^ + 2*F*_c_^2^)/3
*S* = 1.17	(Δ/σ)_max_ = 0.001
6976 reflections	Δρ_max_ = 0.93 e Å^−^^3^
398 parameters	Δρ_min_ = −0.81 e Å^−^^3^
0 restraints	Absolute structure: Flack (1983), 2913 Friedel pairs
Primary atom site location: structure-invariant direct methods	Absolute structure parameter: 0.18 (5)

### Special details

**Table:**

Geometry. All esds (except the esd in the dihedral angle between two l.s. planes) are estimated using the full covariance matrix. The cell esds are taken into account individually in the estimation of esds in distances, angles and torsion angles; correlations between esds in cell parameters are only used when they are defined by crystal symmetry. An approximate (isotropic) treatment of cell esds is used for estimating esds involving l.s. planes.
Refinement. Refined as a 2-component inversion twin.

### Fractional atomic coordinates and isotropic or equivalent isotropic displacement parameters (Å^2^)

**Table:**

	*x*	*y*	*z*	*U*_iso_*/*U*_eq_	
Cu1	0.03847 (9)	0.68690 (8)	−0.03396 (3)	0.0359 (3)	
Cu2	0.19814 (9)	0.54936 (8)	−0.03808 (3)	0.0352 (3)	
Cu3	0.19184 (8)	0.68392 (9)	0.03586 (3)	0.0336 (3)	
Cu4	0.05180 (8)	0.52599 (8)	0.03383 (4)	0.0366 (3)	
Cl1	0.02892 (19)	0.84482 (16)	−0.04415 (7)	0.0529 (6)	
Cl2	0.20776 (19)	0.39762 (16)	−0.05687 (8)	0.0545 (6)	
Cl3	0.34843 (16)	0.68975 (19)	0.04958 (7)	0.0502 (6)	
Cl4	−0.10283 (18)	0.5147 (2)	0.04883 (9)	0.0632 (8)	
N1	0.3352 (5)	0.5899 (5)	−0.05397 (18)	0.0368 (16)	
N2	−0.0955 (6)	0.6526 (5)	−0.0528 (2)	0.0417 (18)	
N3	0.1545 (5)	0.8161 (5)	0.05649 (18)	0.0341 (15)	
N4	0.0914 (5)	0.3890 (5)	0.04787 (19)	0.0361 (16)	
O1	0.1787 (4)	0.6858 (4)	−0.02744 (15)	0.0325 (12)	
O2	0.0590 (4)	0.5475 (4)	−0.02994 (15)	0.0370 (13)	
O3	0.0527 (4)	0.6684 (4)	0.02958 (17)	0.0394 (14)	
O4	0.1893 (4)	0.5442 (4)	0.02558 (15)	0.0346 (12)	
C1	0.2455 (6)	0.7412 (6)	−0.0465 (2)	0.040 (2)	
H1A	0.2478	0.8062	−0.0374	0.048*	
H1B	0.2257	0.7445	−0.0704	0.048*	
C2	0.3414 (6)	0.6967 (6)	−0.0440 (2)	0.0364 (19)	
H2A	0.3656	0.7027	−0.0207	0.044*	
H2B	0.3857	0.7296	−0.0593	0.044*	
C3	0.3439 (7)	0.5788 (7)	−0.0916 (2)	0.042 (2)	
H3A	0.2995	0.6228	−0.1027	0.050*	
H3B	0.3252	0.5139	−0.0979	0.050*	
C4	0.4451 (8)	0.5979 (8)	−0.1053 (3)	0.051 (2)	
H4A	0.4479	0.5813	−0.1295	0.062*	
H4B	0.4597	0.6659	−0.1031	0.062*	
C5	0.5196 (7)	0.5397 (8)	−0.0857 (2)	0.050 (2)	
H5A	0.5836	0.5577	−0.0932	0.060*	
H5B	0.5106	0.4718	−0.0904	0.060*	
C6	0.5095 (5)	0.5583 (8)	−0.0469 (2)	0.043 (2)	
H6A	0.5560	0.5198	−0.0344	0.051*	
H6B	0.5221	0.6255	−0.0420	0.051*	
C7	0.4089 (6)	0.5323 (7)	−0.0357 (2)	0.040 (2)	
H7A	0.3977	0.4646	−0.0401	0.048*	
H7B	0.4028	0.5430	−0.0111	0.048*	
C8	−0.0104 (7)	0.4958 (6)	−0.0498 (3)	0.046 (2)	
H8A	−0.0158	0.4302	−0.0415	0.055*	
H8B	0.0084	0.4944	−0.0738	0.055*	
C9	−0.1069 (6)	0.5489 (6)	−0.0455 (3)	0.041 (2)	
H9A	−0.1540	0.5217	−0.0612	0.050*	
H9B	−0.1304	0.5404	−0.0222	0.050*	
C10	−0.1754 (6)	0.7091 (7)	−0.0363 (3)	0.044 (2)	
H10A	−0.1600	0.7771	−0.0376	0.053*	
H10B	−0.1791	0.6918	−0.0121	0.053*	
C11	−0.2722 (7)	0.6925 (9)	−0.0527 (4)	0.072 (4)	
H11A	−0.3205	0.7310	−0.0411	0.087*	
H11B	−0.2900	0.6253	−0.0504	0.087*	
C12	−0.2676 (9)	0.7200 (9)	−0.0907 (4)	0.074 (4)	
H12A	−0.3292	0.7074	−0.1016	0.089*	
H12B	−0.2537	0.7881	−0.0930	0.089*	
C13	−0.1893 (10)	0.6614 (9)	−0.1083 (3)	0.068 (3)	
H13A	−0.1835	0.6819	−0.1321	0.082*	
H13B	−0.2074	0.5940	−0.1082	0.082*	
C14	−0.0920 (7)	0.6728 (7)	−0.0904 (2)	0.047 (2)	
H14A	−0.0461	0.6296	−0.1011	0.056*	
H14B	−0.0692	0.7380	−0.0939	0.056*	
C15	0.0002 (7)	0.7326 (6)	0.0510 (3)	0.048 (2)	
H15A	−0.0012	0.7091	0.0745	0.058*	
H15B	−0.0654	0.7390	0.0428	0.058*	
C16	0.0504 (7)	0.8256 (7)	0.0492 (3)	0.048 (2)	
H16A	0.0220	0.8695	0.0657	0.057*	
H16B	0.0419	0.8530	0.0264	0.057*	
C17	0.2152 (7)	0.8908 (6)	0.0417 (2)	0.040 (2)	
H17A	0.2016	0.8962	0.0172	0.047*	
H17B	0.2822	0.8726	0.0442	0.047*	
C18	0.1989 (8)	0.9886 (7)	0.0588 (3)	0.055 (3)	
H18A	0.2402	1.0364	0.0482	0.066*	
H18B	0.1326	1.0085	0.0555	0.066*	
C19	0.2207 (8)	0.9825 (8)	0.0970 (3)	0.058 (3)	
H19A	0.2888	0.9703	0.1004	0.069*	
H19B	0.2048	1.0430	0.1080	0.069*	
C20	0.1625 (8)	0.9022 (7)	0.1127 (3)	0.050 (2)	
H20A	0.1810	0.8947	0.1367	0.060*	
H20B	0.0949	0.9196	0.1122	0.060*	
C21	0.1757 (7)	0.8089 (7)	0.0946 (2)	0.041 (2)	
H21A	0.1336	0.7612	0.1048	0.050*	
H21B	0.2415	0.7873	0.0977	0.050*	
C22	0.2464 (7)	0.4756 (7)	0.0441 (3)	0.048 (2)	
H22A	0.2485	0.4913	0.0684	0.057*	
H22B	0.3115	0.4738	0.0352	0.057*	
C23	0.1969 (6)	0.3820 (7)	0.0384 (3)	0.043 (2)	
H23A	0.2274	0.3326	0.0522	0.052*	
H23B	0.2028	0.3637	0.0143	0.052*	
C24	0.0357 (8)	0.3141 (7)	0.0299 (3)	0.051 (2)	
H24A	0.0519	0.3168	0.0056	0.061*	
H24B	−0.0320	0.3298	0.0320	0.061*	
C25	0.0498 (10)	0.2113 (9)	0.0421 (3)	0.068 (3)	
H25A	0.0042	0.1698	0.0304	0.082*	
H25B	0.1141	0.1901	0.0360	0.082*	
C26	0.0364 (8)	0.2022 (7)	0.0800 (3)	0.055 (3)	
H26A	0.0553	0.1381	0.0872	0.066*	
H26B	−0.0310	0.2108	0.0856	0.066*	
C27	0.0955 (9)	0.2757 (8)	0.0991 (3)	0.054 (3)	
H27A	0.1632	0.2599	0.0967	0.065*	
H27B	0.0795	0.2731	0.1234	0.065*	
C28	0.0785 (7)	0.3783 (7)	0.0857 (2)	0.044 (2)	
H28A	0.0137	0.3978	0.0917	0.053*	
H28B	0.1227	0.4216	0.0973	0.053*	

### Atomic displacement parameters (Å^2^)

**Table:**

	*U*^11^	*U*^22^	*U*^33^	*U*^12^	*U*^13^	*U*^23^
Cu1	0.0306 (6)	0.0252 (6)	0.0519 (7)	0.0000 (5)	−0.0094 (6)	0.0002 (6)
Cu2	0.0311 (6)	0.0219 (6)	0.0527 (7)	0.0011 (5)	−0.0025 (6)	−0.0030 (5)
Cu3	0.0254 (6)	0.0291 (6)	0.0463 (7)	0.0015 (5)	−0.0030 (5)	−0.0041 (5)
Cu4	0.0252 (6)	0.0267 (6)	0.0578 (8)	−0.0005 (5)	−0.0042 (6)	0.0029 (6)
Cl1	0.0513 (14)	0.0268 (10)	0.0807 (17)	0.0015 (9)	−0.0163 (12)	0.0049 (11)
Cl2	0.0510 (14)	0.0273 (11)	0.0854 (18)	−0.0012 (10)	0.0042 (12)	−0.0133 (11)
Cl3	0.0267 (10)	0.0533 (14)	0.0706 (15)	0.0043 (10)	−0.0088 (10)	−0.0159 (12)
Cl4	0.0335 (12)	0.0500 (14)	0.106 (2)	−0.0035 (10)	0.0059 (13)	0.0126 (14)
N1	0.038 (4)	0.036 (4)	0.036 (4)	−0.010 (3)	−0.003 (3)	−0.001 (3)
N2	0.043 (4)	0.027 (4)	0.055 (4)	−0.007 (3)	−0.018 (4)	0.002 (3)
N3	0.043 (4)	0.021 (3)	0.038 (4)	0.000 (3)	−0.002 (3)	−0.004 (3)
N4	0.026 (4)	0.034 (4)	0.048 (4)	−0.008 (3)	−0.001 (3)	0.002 (3)
O1	0.025 (3)	0.025 (3)	0.047 (3)	0.008 (2)	−0.003 (2)	0.001 (2)
O2	0.031 (3)	0.026 (3)	0.054 (3)	0.010 (2)	−0.011 (3)	−0.008 (3)
O3	0.025 (3)	0.026 (3)	0.067 (4)	0.009 (2)	0.002 (3)	−0.005 (3)
O4	0.028 (3)	0.026 (3)	0.050 (3)	0.008 (2)	−0.005 (2)	0.005 (2)
C1	0.034 (5)	0.027 (4)	0.058 (5)	−0.004 (3)	0.022 (4)	0.003 (4)
C2	0.026 (4)	0.039 (5)	0.044 (5)	−0.006 (4)	0.002 (3)	−0.011 (4)
C3	0.038 (5)	0.039 (5)	0.049 (5)	0.006 (4)	−0.002 (4)	−0.002 (4)
C4	0.058 (6)	0.050 (6)	0.046 (5)	0.000 (5)	0.008 (5)	0.005 (4)
C5	0.042 (5)	0.057 (6)	0.052 (6)	0.011 (5)	0.005 (4)	−0.008 (5)
C6	0.010 (4)	0.053 (6)	0.064 (6)	0.005 (3)	0.001 (4)	0.013 (5)
C7	0.024 (4)	0.049 (5)	0.048 (5)	−0.009 (4)	0.004 (4)	0.005 (4)
C8	0.043 (5)	0.023 (4)	0.071 (6)	−0.004 (4)	−0.022 (5)	−0.002 (4)
C9	0.027 (4)	0.028 (4)	0.068 (6)	0.006 (3)	−0.021 (4)	0.005 (4)
C10	0.021 (4)	0.039 (5)	0.072 (6)	0.003 (3)	0.001 (4)	−0.008 (5)
C11	0.032 (5)	0.063 (7)	0.121 (11)	0.027 (5)	−0.018 (6)	−0.035 (7)
C12	0.058 (7)	0.049 (6)	0.115 (11)	0.015 (5)	−0.044 (7)	0.003 (7)
C13	0.083 (9)	0.053 (7)	0.068 (7)	−0.001 (6)	−0.037 (7)	0.004 (5)
C14	0.053 (6)	0.038 (5)	0.050 (5)	−0.005 (4)	−0.008 (4)	0.002 (4)
C15	0.038 (5)	0.026 (4)	0.080 (7)	0.003 (4)	−0.002 (5)	−0.012 (5)
C16	0.047 (5)	0.034 (5)	0.062 (6)	0.023 (4)	−0.010 (5)	−0.020 (4)
C17	0.052 (6)	0.029 (4)	0.038 (5)	0.005 (4)	0.012 (4)	−0.002 (4)
C18	0.049 (6)	0.034 (5)	0.083 (7)	−0.010 (4)	0.003 (5)	0.005 (5)
C19	0.060 (6)	0.048 (6)	0.065 (6)	−0.005 (5)	−0.005 (5)	−0.022 (5)
C20	0.057 (6)	0.047 (6)	0.046 (5)	−0.008 (5)	−0.004 (5)	−0.016 (4)
C21	0.046 (5)	0.041 (5)	0.037 (4)	−0.008 (4)	0.000 (4)	−0.001 (4)
C22	0.030 (5)	0.040 (5)	0.073 (7)	0.013 (4)	−0.012 (4)	0.010 (5)
C23	0.021 (4)	0.048 (5)	0.060 (6)	0.018 (4)	0.007 (4)	0.007 (4)
C24	0.055 (6)	0.046 (5)	0.051 (5)	−0.011 (5)	−0.004 (5)	0.004 (5)
C25	0.065 (8)	0.069 (8)	0.070 (7)	0.034 (6)	0.013 (6)	0.001 (6)
C26	0.048 (6)	0.043 (6)	0.073 (7)	0.009 (5)	0.004 (5)	0.016 (5)
C27	0.071 (7)	0.044 (6)	0.048 (6)	0.006 (5)	−0.004 (5)	0.013 (4)
C28	0.048 (5)	0.038 (5)	0.046 (5)	−0.001 (4)	−0.009 (4)	−0.009 (4)

### Geometric parameters (Å, º)

**Table:**

Cu1—O2	1.965 (6)	C8—H8B	0.9700
Cu1—O1	1.965 (5)	C9—H9A	0.9700
Cu1—N2	2.058 (7)	C9—H9B	0.9700
Cu1—Cl1	2.235 (2)	C10—C11	1.507 (13)
Cu1—Cu2	2.9341 (17)	C10—H10A	0.9700
Cu2—O2	1.960 (6)	C10—H10B	0.9700
Cu2—O1	1.960 (5)	C11—C12	1.529 (19)
Cu2—N1	2.081 (7)	C11—H11A	0.9700
Cu2—Cl2	2.236 (2)	C11—H11B	0.9700
Cu3—O3	1.961 (6)	C12—C13	1.522 (18)
Cu3—O4	1.983 (6)	C12—H12A	0.9700
Cu3—N3	2.070 (7)	C12—H12B	0.9700
Cu3—Cl3	2.242 (2)	C13—C14	1.528 (15)
Cu3—Cu4	2.9354 (17)	C13—H13A	0.9700
Cu4—O4	1.954 (6)	C13—H13B	0.9700
Cu4—O3	1.986 (6)	C14—H14A	0.9700
Cu4—N4	2.057 (7)	C14—H14B	0.9700
Cu4—Cl4	2.233 (3)	C15—C16	1.472 (13)
N1—C3	1.476 (11)	C15—H15A	0.9700
N1—C7	1.482 (12)	C15—H15B	0.9700
N1—C2	1.536 (11)	C16—H16A	0.9700
N2—C9	1.478 (11)	C16—H16B	0.9700
N2—C14	1.488 (12)	C17—C18	1.532 (13)
N2—C10	1.505 (12)	C17—H17A	0.9700
N3—C17	1.457 (11)	C17—H17B	0.9700
N3—C16	1.480 (12)	C18—C19	1.514 (15)
N3—C21	1.512 (11)	C18—H18A	0.9700
N4—C24	1.474 (12)	C18—H18B	0.9700
N4—C28	1.488 (12)	C19—C20	1.508 (15)
N4—C23	1.515 (10)	C19—H19A	0.9700
O1—C1	1.416 (9)	C19—H19B	0.9700
O2—C8	1.429 (10)	C20—C21	1.488 (13)
O3—C15	1.421 (11)	C20—H20A	0.9700
O4—C22	1.434 (10)	C20—H20B	0.9700
C1—C2	1.473 (12)	C21—H21A	0.9700
C1—H1A	0.9700	C21—H21B	0.9700
C1—H1B	0.9700	C22—C23	1.489 (14)
C2—H2A	0.9700	C22—H22A	0.9700
C2—H2B	0.9700	C22—H22B	0.9700
C3—C4	1.526 (14)	C23—H23A	0.9700
C3—H3A	0.9700	C23—H23B	0.9700
C3—H3B	0.9700	C24—C25	1.518 (15)
C4—C5	1.519 (14)	C24—H24A	0.9700
C4—H4A	0.9700	C24—H24B	0.9700
C4—H4B	0.9700	C25—C26	1.488 (15)
C5—C6	1.533 (13)	C25—H25A	0.9700
C5—H5A	0.9700	C25—H25B	0.9700
C5—H5B	0.9700	C26—C27	1.508 (15)
C6—C7	1.510 (11)	C26—H26A	0.9700
C6—H6A	0.9700	C26—H26B	0.9700
C6—H6B	0.9700	C27—C28	1.536 (13)
C7—H7A	0.9700	C27—H27A	0.9700
C7—H7B	0.9700	C27—H27B	0.9700
C8—C9	1.540 (12)	C28—H28A	0.9700
C8—H8A	0.9700	C28—H28B	0.9700
			
O2—Cu1—O1	80.7 (2)	C9—C8—H8B	110.4
O2—Cu1—N2	86.1 (3)	H8A—C8—H8B	108.6
O1—Cu1—N2	160.3 (3)	N2—C9—C8	110.7 (7)
O2—Cu1—Cl1	172.5 (2)	N2—C9—H9A	109.5
O1—Cu1—Cl1	95.13 (18)	C8—C9—H9A	109.5
N2—Cu1—Cl1	96.4 (2)	N2—C9—H9B	109.5
O2—Cu1—Cu2	41.55 (17)	C8—C9—H9B	109.5
O1—Cu1—Cu2	41.56 (16)	H9A—C9—H9B	108.1
N2—Cu1—Cu2	121.0 (2)	N2—C10—C11	113.4 (8)
Cl1—Cu1—Cu2	132.50 (9)	N2—C10—H10A	108.9
O2—Cu2—O1	80.9 (2)	C11—C10—H10A	108.9
O2—Cu2—N1	162.8 (3)	N2—C10—H10B	108.9
O1—Cu2—N1	85.8 (3)	C11—C10—H10B	108.9
O2—Cu2—Cl2	95.71 (18)	H10A—C10—H10B	107.7
O1—Cu2—Cl2	171.88 (19)	C10—C11—C12	109.5 (11)
N1—Cu2—Cl2	96.0 (2)	C10—C11—H11A	109.8
O2—Cu2—Cu1	41.67 (16)	C12—C11—H11A	109.8
O1—Cu2—Cu1	41.69 (16)	C10—C11—H11B	109.8
N1—Cu2—Cu1	122.2 (2)	C12—C11—H11B	109.8
Cl2—Cu2—Cu1	132.67 (9)	H11A—C11—H11B	108.2
O3—Cu3—O4	81.3 (2)	C13—C12—C11	109.2 (9)
O3—Cu3—N3	84.2 (3)	C13—C12—H12A	109.8
O4—Cu3—N3	160.5 (3)	C11—C12—H12A	109.8
O3—Cu3—Cl3	172.2 (2)	C13—C12—H12B	109.8
O4—Cu3—Cl3	95.78 (18)	C11—C12—H12B	109.8
N3—Cu3—Cl3	96.9 (2)	H12A—C12—H12B	108.3
O3—Cu3—Cu4	42.29 (16)	C12—C13—C14	112.0 (9)
O4—Cu3—Cu4	41.42 (17)	C12—C13—H13A	109.2
N3—Cu3—Cu4	120.5 (2)	C14—C13—H13A	109.2
Cl3—Cu3—Cu4	132.62 (9)	C12—C13—H13B	109.2
O4—Cu4—O3	81.4 (2)	C14—C13—H13B	109.2
O4—Cu4—N4	84.4 (2)	H13A—C13—H13B	107.9
O3—Cu4—N4	160.6 (3)	N2—C14—C13	113.4 (9)
O4—Cu4—Cl4	173.4 (2)	N2—C14—H14A	108.9
O3—Cu4—Cl4	95.61 (18)	C13—C14—H14A	108.9
N4—Cu4—Cl4	97.1 (2)	N2—C14—H14B	108.9
O4—Cu4—Cu3	42.17 (16)	C13—C14—H14B	108.9
O3—Cu4—Cu3	41.63 (16)	H14A—C14—H14B	107.7
N4—Cu4—Cu3	120.5 (2)	O3—C15—C16	106.3 (8)
Cl4—Cu4—Cu3	133.15 (9)	O3—C15—H15A	110.5
C3—N1—C7	111.3 (7)	C16—C15—H15A	110.5
C3—N1—C2	110.2 (7)	O3—C15—H15B	110.5
C7—N1—C2	111.3 (7)	C16—C15—H15B	110.5
C3—N1—Cu2	109.9 (5)	H15A—C15—H15B	108.7
C7—N1—Cu2	110.1 (5)	C15—C16—N3	112.1 (7)
C2—N1—Cu2	103.8 (5)	C15—C16—H16A	109.2
C9—N2—C14	112.1 (7)	N3—C16—H16A	109.2
C9—N2—C10	110.3 (7)	C15—C16—H16B	109.2
C14—N2—C10	110.2 (7)	N3—C16—H16B	109.2
C9—N2—Cu1	104.8 (5)	H16A—C16—H16B	107.9
C14—N2—Cu1	106.1 (6)	N3—C17—C18	112.0 (7)
C10—N2—Cu1	113.2 (5)	N3—C17—H17A	109.2
C17—N3—C16	115.2 (7)	C18—C17—H17A	109.2
C17—N3—C21	108.7 (7)	N3—C17—H17B	109.2
C16—N3—C21	112.6 (7)	C18—C17—H17B	109.2
C17—N3—Cu3	109.6 (5)	H17A—C17—H17B	107.9
C16—N3—Cu3	104.5 (5)	C19—C18—C17	110.3 (9)
C21—N3—Cu3	105.7 (5)	C19—C18—H18A	109.6
C24—N4—C28	109.5 (7)	C17—C18—H18A	109.6
C24—N4—C23	110.4 (8)	C19—C18—H18B	109.6
C28—N4—C23	110.6 (7)	C17—C18—H18B	109.6
C24—N4—Cu4	112.8 (6)	H18A—C18—H18B	108.1
C28—N4—Cu4	108.8 (5)	C20—C19—C18	109.3 (8)
C23—N4—Cu4	104.7 (5)	C20—C19—H19A	109.8
C1—O1—Cu2	109.0 (5)	C18—C19—H19A	109.8
C1—O1—Cu1	125.4 (5)	C20—C19—H19B	109.8
Cu2—O1—Cu1	96.7 (2)	C18—C19—H19B	109.8
C8—O2—Cu2	125.9 (6)	H19A—C19—H19B	108.3
C8—O2—Cu1	110.8 (5)	C21—C20—C19	112.7 (9)
Cu2—O2—Cu1	96.8 (3)	C21—C20—H20A	109.1
C15—O3—Cu3	111.4 (5)	C19—C20—H20A	109.1
C15—O3—Cu4	125.0 (6)	C21—C20—H20B	109.1
Cu3—O3—Cu4	96.1 (2)	C19—C20—H20B	109.1
C22—O4—Cu4	111.9 (5)	H20A—C20—H20B	107.8
C22—O4—Cu3	122.7 (6)	C20—C21—N3	112.5 (8)
Cu4—O4—Cu3	96.4 (2)	C20—C21—H21A	109.1
O1—C1—C2	109.4 (7)	N3—C21—H21A	109.1
O1—C1—H1A	109.8	C20—C21—H21B	109.1
C2—C1—H1A	109.8	N3—C21—H21B	109.1
O1—C1—H1B	109.8	H21A—C21—H21B	107.8
C2—C1—H1B	109.8	O4—C22—C23	104.5 (7)
H1A—C1—H1B	108.2	O4—C22—H22A	110.8
C1—C2—N1	109.8 (7)	C23—C22—H22A	110.8
C1—C2—H2A	109.7	O4—C22—H22B	110.8
N1—C2—H2A	109.7	C23—C22—H22B	110.8
C1—C2—H2B	109.7	H22A—C22—H22B	108.9
N1—C2—H2B	109.7	C22—C23—N4	110.8 (7)
H2A—C2—H2B	108.2	C22—C23—H23A	109.5
N1—C3—C4	113.6 (8)	N4—C23—H23A	109.5
N1—C3—H3A	108.8	C22—C23—H23B	109.5
C4—C3—H3A	108.8	N4—C23—H23B	109.5
N1—C3—H3B	108.8	H23A—C23—H23B	108.1
C4—C3—H3B	108.8	N4—C24—C25	116.7 (9)
H3A—C3—H3B	107.7	N4—C24—H24A	108.1
C5—C4—C3	111.2 (8)	C25—C24—H24A	108.1
C5—C4—H4A	109.4	N4—C24—H24B	108.1
C3—C4—H4A	109.4	C25—C24—H24B	108.1
C5—C4—H4B	109.4	H24A—C24—H24B	107.3
C3—C4—H4B	109.4	C26—C25—C24	111.9 (9)
H4A—C4—H4B	108.0	C26—C25—H25A	109.2
C4—C5—C6	109.9 (8)	C24—C25—H25A	109.2
C4—C5—H5A	109.7	C26—C25—H25B	109.2
C6—C5—H5A	109.7	C24—C25—H25B	109.2
C4—C5—H5B	109.7	H25A—C25—H25B	107.9
C6—C5—H5B	109.7	C25—C26—C27	111.1 (10)
H5A—C5—H5B	108.2	C25—C26—H26A	109.4
C7—C6—C5	109.2 (8)	C27—C26—H26A	109.4
C7—C6—H6A	109.8	C25—C26—H26B	109.4
C5—C6—H6A	109.8	C27—C26—H26B	109.4
C7—C6—H6B	109.8	H26A—C26—H26B	108.0
C5—C6—H6B	109.8	C26—C27—C28	112.3 (8)
H6A—C6—H6B	108.3	C26—C27—H27A	109.1
N1—C7—C6	111.9 (7)	C28—C27—H27A	109.1
N1—C7—H7A	109.2	C26—C27—H27B	109.1
C6—C7—H7A	109.2	C28—C27—H27B	109.1
N1—C7—H7B	109.2	H27A—C27—H27B	107.9
C6—C7—H7B	109.2	N4—C28—C27	114.0 (8)
H7A—C7—H7B	107.9	N4—C28—H28A	108.7
O2—C8—C9	106.9 (7)	C27—C28—H28A	108.7
O2—C8—H8A	110.4	N4—C28—H28B	108.7
C9—C8—H8A	110.4	C27—C28—H28B	108.7
O2—C8—H8B	110.4	H28A—C28—H28B	107.6
			
O1—Cu1—Cu2—O2	154.7 (4)	N2—Cu1—O2—C8	16.2 (6)
N2—Cu1—Cu2—O2	−37.2 (4)	Cl1—Cu1—O2—C8	−93.1 (16)
Cl1—Cu1—Cu2—O2	−173.6 (3)	Cu2—Cu1—O2—C8	−132.6 (7)
O2—Cu1—Cu2—O1	−154.7 (4)	O1—Cu1—O2—Cu2	−16.7 (2)
N2—Cu1—Cu2—O1	168.2 (4)	N2—Cu1—O2—Cu2	148.7 (3)
Cl1—Cu1—Cu2—O1	31.7 (3)	Cl1—Cu1—O2—Cu2	39.4 (16)
O2—Cu1—Cu2—N1	172.0 (3)	O4—Cu3—O3—C15	147.7 (6)
O1—Cu1—Cu2—N1	−33.3 (3)	N3—Cu3—O3—C15	−19.1 (6)
N2—Cu1—Cu2—N1	134.9 (4)	Cl3—Cu3—O3—C15	79.0 (15)
Cl1—Cu1—Cu2—N1	−1.5 (3)	Cu4—Cu3—O3—C15	131.4 (7)
O2—Cu1—Cu2—Cl2	33.7 (3)	O4—Cu3—O3—Cu4	16.3 (2)
O1—Cu1—Cu2—Cl2	−171.6 (3)	N3—Cu3—O3—Cu4	−150.5 (3)
N2—Cu1—Cu2—Cl2	−3.5 (3)	Cl3—Cu3—O3—Cu4	−52.4 (15)
Cl1—Cu1—Cu2—Cl2	−139.90 (16)	O4—Cu4—O3—C15	−138.1 (7)
O3—Cu3—Cu4—O4	155.2 (4)	N4—Cu4—O3—C15	−94.8 (10)
N3—Cu3—Cu4—O4	−170.2 (3)	Cl4—Cu4—O3—C15	36.0 (7)
Cl3—Cu3—Cu4—O4	−33.2 (3)	Cu3—Cu4—O3—C15	−121.5 (8)
O4—Cu3—Cu4—O3	−155.2 (4)	O4—Cu4—O3—Cu3	−16.6 (3)
N3—Cu3—Cu4—O3	34.6 (4)	N4—Cu4—O3—Cu3	26.6 (10)
Cl3—Cu3—Cu4—O3	171.6 (3)	Cl4—Cu4—O3—Cu3	157.5 (2)
O3—Cu3—Cu4—N4	−170.0 (4)	O3—Cu4—O4—C22	145.5 (6)
O4—Cu3—Cu4—N4	34.8 (3)	N4—Cu4—O4—C22	−21.3 (6)
N3—Cu3—Cu4—N4	−135.4 (3)	Cl4—Cu4—O4—C22	82.0 (17)
Cl3—Cu3—Cu4—N4	1.6 (3)	Cu3—Cu4—O4—C22	129.1 (7)
O3—Cu3—Cu4—Cl4	−31.5 (3)	O3—Cu4—O4—Cu3	16.4 (2)
O4—Cu3—Cu4—Cl4	173.4 (3)	N4—Cu4—O4—Cu3	−150.4 (3)
N3—Cu3—Cu4—Cl4	3.2 (3)	Cl4—Cu4—O4—Cu3	−47.1 (17)
Cl3—Cu3—Cu4—Cl4	140.15 (16)	O3—Cu3—O4—C22	−137.8 (7)
O2—Cu2—N1—C3	−72.1 (11)	N3—Cu3—O4—C22	−95.2 (10)
O1—Cu2—N1—C3	−111.7 (6)	Cl3—Cu3—O4—C22	34.9 (7)
Cl2—Cu2—N1—C3	60.4 (6)	Cu4—Cu3—O4—C22	−121.2 (7)
Cu1—Cu2—N1—C3	−90.2 (6)	O3—Cu3—O4—Cu4	−16.6 (2)
O2—Cu2—N1—C7	165.0 (8)	N3—Cu3—O4—Cu4	26.0 (9)
O1—Cu2—N1—C7	125.4 (6)	Cl3—Cu3—O4—Cu4	156.08 (19)
Cl2—Cu2—N1—C7	−62.6 (6)	Cu2—O1—C1—C2	−45.4 (8)
Cu1—Cu2—N1—C7	146.9 (5)	Cu1—O1—C1—C2	−158.8 (5)
O2—Cu2—N1—C2	45.8 (11)	O1—C1—C2—N1	52.2 (10)
O1—Cu2—N1—C2	6.2 (5)	C3—N1—C2—C1	85.9 (9)
Cl2—Cu2—N1—C2	178.2 (5)	C7—N1—C2—C1	−150.2 (7)
Cu1—Cu2—N1—C2	27.6 (6)	Cu2—N1—C2—C1	−31.7 (8)
O2—Cu1—N2—C9	11.8 (6)	C7—N1—C3—C4	−52.3 (10)
O1—Cu1—N2—C9	59.4 (11)	C2—N1—C3—C4	71.6 (10)
Cl1—Cu1—N2—C9	−175.2 (6)	Cu2—N1—C3—C4	−174.6 (6)
Cu2—Cu1—N2—C9	35.5 (7)	N1—C3—C4—C5	51.6 (11)
O2—Cu1—N2—C14	−106.9 (6)	C3—C4—C5—C6	−53.8 (12)
O1—Cu1—N2—C14	−59.4 (10)	C4—C5—C6—C7	58.0 (11)
Cl1—Cu1—N2—C14	66.0 (6)	C3—N1—C7—C6	56.8 (10)
Cu2—Cu1—N2—C14	−83.2 (6)	C2—N1—C7—C6	−66.5 (10)
O2—Cu1—N2—C10	132.1 (6)	Cu2—N1—C7—C6	179.0 (7)
O1—Cu1—N2—C10	179.7 (7)	C5—C6—C7—N1	−59.9 (10)
Cl1—Cu1—N2—C10	−55.0 (6)	Cu2—O2—C8—C9	−154.7 (6)
Cu2—Cu1—N2—C10	155.8 (5)	Cu1—O2—C8—C9	−39.2 (9)
O3—Cu3—N3—C17	−132.6 (6)	C14—N2—C9—C8	78.3 (9)
O4—Cu3—N3—C17	−174.8 (7)	C10—N2—C9—C8	−158.4 (8)
Cl3—Cu3—N3—C17	55.2 (6)	Cu1—N2—C9—C8	−36.2 (9)
Cu4—Cu3—N3—C17	−155.2 (5)	O2—C8—C9—N2	51.1 (10)
O3—Cu3—N3—C16	−8.6 (6)	C9—N2—C10—C11	−69.4 (11)
O4—Cu3—N3—C16	−50.8 (11)	C14—N2—C10—C11	54.9 (11)
Cl3—Cu3—N3—C16	179.2 (6)	Cu1—N2—C10—C11	173.6 (8)
Cu4—Cu3—N3—C16	−31.2 (6)	N2—C10—C11—C12	−58.9 (12)
O3—Cu3—N3—C21	110.4 (6)	C10—C11—C12—C13	57.6 (12)
O4—Cu3—N3—C21	68.2 (10)	C11—C12—C13—C14	−55.2 (13)
Cl3—Cu3—N3—C21	−61.8 (5)	C9—N2—C14—C13	72.3 (10)
Cu4—Cu3—N3—C21	87.8 (5)	C10—N2—C14—C13	−51.0 (10)
O4—Cu4—N4—C24	−128.1 (6)	Cu1—N2—C14—C13	−173.9 (7)
O3—Cu4—N4—C24	−171.0 (7)	C12—C13—C14—N2	53.1 (12)
Cl4—Cu4—N4—C24	58.3 (6)	Cu3—O3—C15—C16	42.3 (9)
Cu3—Cu4—N4—C24	−150.8 (5)	Cu4—O3—C15—C16	156.7 (6)
O4—Cu4—N4—C28	110.1 (6)	O3—C15—C16—N3	−51.6 (11)
O3—Cu4—N4—C28	67.2 (10)	C17—N3—C16—C15	155.0 (8)
Cl4—Cu4—N4—C28	−63.4 (6)	C21—N3—C16—C15	−79.6 (10)
Cu3—Cu4—N4—C28	87.5 (5)	Cu3—N3—C16—C15	34.7 (10)
O4—Cu4—N4—C23	−8.1 (6)	C16—N3—C17—C18	69.0 (10)
O3—Cu4—N4—C23	−51.0 (11)	C21—N3—C17—C18	−58.5 (10)
Cl4—Cu4—N4—C23	178.4 (5)	Cu3—N3—C17—C18	−173.6 (7)
Cu3—Cu4—N4—C23	−30.7 (6)	N3—C17—C18—C19	59.5 (11)
O2—Cu2—O1—C1	−147.9 (6)	C17—C18—C19—C20	−54.4 (12)
N1—Cu2—O1—C1	21.1 (6)	C18—C19—C20—C21	53.5 (13)
Cl2—Cu2—O1—C1	−82.0 (15)	C19—C20—C21—N3	−55.0 (12)
Cu1—Cu2—O1—C1	−131.2 (6)	C17—N3—C21—C20	56.3 (10)
O2—Cu2—O1—Cu1	−16.7 (2)	C16—N3—C21—C20	−72.6 (10)
N1—Cu2—O1—Cu1	152.2 (3)	Cu3—N3—C21—C20	173.9 (7)
Cl2—Cu2—O1—Cu1	49.2 (15)	Cu4—O4—C22—C23	45.1 (9)
O2—Cu1—O1—C1	135.8 (6)	Cu3—O4—C22—C23	158.7 (6)
N2—Cu1—O1—C1	87.6 (10)	O4—C22—C23—N4	−53.0 (10)
Cl1—Cu1—O1—C1	−38.0 (6)	C24—N4—C23—C22	157.2 (8)
Cu2—Cu1—O1—C1	119.1 (7)	C28—N4—C23—C22	−81.5 (9)
O2—Cu1—O1—Cu2	16.7 (2)	Cu4—N4—C23—C22	35.5 (9)
N2—Cu1—O1—Cu2	−31.5 (9)	C28—N4—C24—C25	−50.4 (11)
Cl1—Cu1—O1—Cu2	−157.1 (2)	C23—N4—C24—C25	71.5 (11)
O1—Cu2—O2—C8	138.5 (7)	Cu4—N4—C24—C25	−171.7 (7)
N1—Cu2—O2—C8	98.5 (10)	N4—C24—C25—C26	52.0 (14)
Cl2—Cu2—O2—C8	−34.0 (6)	C24—C25—C26—C27	−50.6 (13)
Cu1—Cu2—O2—C8	121.8 (7)	C25—C26—C27—C28	52.0 (12)
O1—Cu2—O2—Cu1	16.7 (2)	C24—N4—C28—C27	50.2 (11)
N1—Cu2—O2—Cu1	−23.3 (10)	C23—N4—C28—C27	−71.7 (10)
Cl2—Cu2—O2—Cu1	−155.8 (2)	Cu4—N4—C28—C27	173.9 (7)
O1—Cu1—O2—C8	−149.3 (6)	C26—C27—C28—N4	−53.0 (12)

### Hydrogen-bond geometry (Å, º)

**Table:**

*D*—H···*A*	*D*—H	H···*A*	*D*···*A*	*D*—H···*A*
C2—H2*A*···Cl3	0.97	2.75	3.638 (9)	153
C2—H2*B*···Cl4^i^	0.97	2.97	3.689 (9)	132
C3—H3*B*···Cl2	0.97	2.80	3.428 (10)	124
C7—H7*A*···Cl2	0.97	2.87	3.464 (9)	120
C9—H9*B*···Cl4	0.97	2.81	3.696 (11)	153
C10—H10*A*···Cl1	0.97	2.80	3.423 (9)	123
C14—H14*B*···Cl1	0.97	2.79	3.432 (10)	124
C16—H16*B*···Cl1	0.97	2.75	3.647 (11)	154
C17—H17*B*···Cl3	0.97	2.71	3.367 (9)	125
C21—H21*B*···Cl3	0.97	2.75	3.400 (10)	125
C23—H23*A*···Cl2^ii^	0.97	2.94	3.765 (9)	144
C23—H23*B*···Cl2	0.97	2.81	3.708 (10)	155
C24—H24*B*···Cl4	0.97	2.83	3.469 (11)	124
C28—H28*A*···Cl4	0.97	2.84	3.465 (10)	123

Symmetry codes: (i) *y*, *x*+1, −*z*; (ii) *y*, *x*, −*z*.
